# mHealth for the Monitoring of Brace Compliance and Wellbeing in Adolescents with Idiopathic Scoliosis: Study Protocol for a Feasibility Study

**DOI:** 10.3390/ijerph18157767

**Published:** 2021-07-22

**Authors:** Verónica Martínez-Borba, Carlos Suso-Ribera, Amanda Díaz-García, Judith Salat-Batlle, Diana Castilla, Irene Zaragoza, Azucena García-Palacios, Judit Sánchez-Raya

**Affiliations:** 1 Instituto de Investigación Sanitaria de Aragón, 50009 Zaragoza, Spain; borba@uji.es; 2 Department of Basic and Clinical Psychology and Psychobiology, Universitat Jaume I, 12071 Castellón, Spain; azucena@uji.es; 3 Department of Psychology and Sociology, Universidad de Zaragoza, 44003 Teruel, Spain; amandadiaz@unizar.es; 4 Physical Medicine and Rehabilitation Service, Hospital Universitario Vall d’Hebron, 08035 Barcelona, Spain; judith.salat@vhir.org (J.S.-B.); jusanchez@vhebron.net (J.S.-R.); 5 Department of Personality, Assessment, and Psychological Treatments, Universidad de Valencia, 46010 Valencia, Spain; diana.castilla@uv.es; 6 CIBER of Physiopathology of Obesity and Nutrition (CIBERON), 28029 Madrid, Spain; irenezaragoza@gmail.com

**Keywords:** adolescent idiopathic scoliosis, Information and Communication Technologies, mobile application, brace adherence, treatment effectiveness

## Abstract

Attempts to optimize monitoring of brace adherence prescribed to adolescents with idiopathic scoliosis (IS) have generally relied on sensors. Sensors, however, are intrusive and do not allow the assessment of psychological and physical consequences of brace use that might underlie poor adherence. Mobile applications have emerged as alternatives to monitor brace compliance. However, the feasibility and utility of these app-based systems to assess key psychological and physical domains associated with non-adherence remain unexplored. This feasibility study aims to test the usability, acceptability, and clinical utility of an app-based system that monitors brace use and related psychological and physical factors. Forty adolescents with IS daily respond to the app for 90 days. The patient responses may generate clinical alarms (e.g., brace non-adherence, discomfort, or distress) that will be sent daily to the medical team. Primary outcomes will be app usability, acceptability, and response rates. Secondary outcomes will include brace adherence, the number of side effects reported, number and type of clinical alarms, stress, quality of life, perceived health status, and mood. If accepted by patients and clinicians, apps may allow rapid detection and response to undesired events in adolescents undergoing brace treatment.

## 1. Introduction

Scoliosis is defined as a torsional deformity in the shape and position of the spine, the thorax, and the trunk, which is generally diagnosed when the Cobb angle, a measure of the spinal deformity, is equal to or higher than 10° [1]. In particular, idiopathic scoliosis (IS) refers to scoliosis that occurs in apparently healthy patients without a clear agent causing the deformity [1]. IS affects 2–3% of adolescents, although it can also occur in newborns. According to the age at which the diagnosis is made, IS can be classified into infantile (0–2 years old), juvenile (3–9 years old), adolescent (Adolescent Idiopathic Scoliosis, AIS; 10–17 years old), or adult (from the age of 18) [1]. The most notable progression of IS occurs at the beginning of puberty (11–14 years), which has been associated with the faster development of the spinal curvature that takes place in this period [1]. In addition to age, IS can also be classified according to the severity of the deformity, measured with the Cobb’s degrees (Low = up to 20°; Moderate = 21°–35°; Moderate-severe = 36°–40°; Severe = 41°–50°; Severe-very severe = 51°–55°; Very severe = over 56°) [1].

IS is often unrelated to medical complications (e.g., pain or other health-related problems) [2,3]. However, even in less severe curves, the deformity can negatively impact the psychological wellbeing of individuals (e.g., depression symptoms and self-esteem) and can be associated with an increase in psychological problems, such as substance abuse and suicidal ideation [4,5], which justifies the need for adequate treatment. Wearing a brace is a conservative treatment for IS that often helps avoid surgery, improves aesthetics, and increases quality of life [1]. Brace treatment consists of the application of an external mechanical force, the objective of which is to correct the spinal deformity. The brace exerts compression forces on the convex side, distraction forces on the concave side, transverse forces on both sides, and lateral bending on the convex side [6]. The brace is especially recommended when the curvature is between 30° and 40° or between 20° and 29° with a progression of more than 5° in the last year [7]. In fact, when scoliosis angles are greater than 30°, the deformity becomes evident and disability, pain, and functional limitations increase significantly [1,8]. Next, depending on the severity of the case, the brace can be prescribed only at night (8–12 h a day), part-time (12–20 h a day), or full time (20–24 h a day) [1].

Review studies have revealed promising results regarding the effectiveness of braces in treating AIS. However, there is insufficient scientific evidence to recommend their use [9], probably due to the low adherence rates associated with brace use, which tend to be exaggerated when retrospectively evaluated [1]. Adherence to the use of the brace is in fact one of the most important factors negatively influencing the effectiveness of this orthopedic treatment [10]. Two factors that have been argued to explain non-compliance with these orthopedic treatments are (a) the appearance of unwanted effects associated with the use of braces (e.g., pain, scuffing, or discomfort sleeping) and (b) the psychological distress and general beliefs related to the deformity (i.e., “I don’t care about my back”) and its treatment (i.e., “I think people can see the brace under my clothes”) [11,12]. Different efforts have been conducted to improve the adherence to the brace. For example, brace monitoring has been proposed to improve brace compliance [13]. For this reason, it has been argued that there is a need to actively involve patients during the whole treatment process [14], including monitoring of outcomes and brace compliance [1].

Traditionally, brace adherence has been explored retrospectively [15]. This is problematic because it does not guarantee the security of the treatment, it does not provide reliable data, and it is an inefficient methodology. First, retrospective assessments do not allow the detection of undesired events in the real context and when they occur, but later when patients have a face-to-face medical appointment. As a consequence, the patient must decide what to do when they experience unwanted side effects [16], which compromises the safety of the intervention. For example, an adolescent may decide to immediately stop wearing the brace when they experience pain or itching and therefore would not receive treatment until the next appointment at the clinic (which can occur weeks or months later, depending on waiting lists). Conversely, another patient may decide to tolerate the discomfort and continue wearing the brace despite unwanted side effects until the next appointment with their doctor, which can lead to undue suffering. Therefore, leaving the decision on when and in the presence of which symptom adolescents should respond to and how they should respond can be problematic. Regarding the second point, which refers to the reliability of the data, research has shown that retrospective assessments are not free from recall bias. In particular, more severe symptoms tend to be reported when health conditions are retrospectively assessed [17]. In the specific case of brace monitoring, it is unlikely that patients will be able to remember the exact number of hours and days that they have been wearing the brace for a given period of time. It is also unlikely that adolescents will be able to remember all the side effects they experienced in the different contexts in which they occurred. The latter point refers to treatment efficiency. Retrospective evaluations are not efficient as they do not allow early detection of noncompliance with brace treatment and psychological distress related to brace use (i.e., irrational beliefs, intense unpleasant emotions, or lack of social support). Therefore, early interventions cannot be delivered in response to these, which means that the patients’ quality of life may be affected. This, in turn, could result in more lasting and severe symptoms (i.e., brace-related side effects, anxiety, and depressive symptoms), ultimately resulting in higher personal and financial costs (i.e., recurrent visits and contacts with health services).

The electronic monitoring of brace compliance has emerged as a possible solution to overcome the aforementioned shortcomings found in IS monitoring. The most commonly used devices to assess brace compliance are temperature and force sensors [13]. Some promising findings have already been reported with these devices. For example, patients are more likely to wear the brace when they know that they are being monitored to avoid professional criticism [18] and sensors have been described as non-invasive [19]. While acknowledging this, it has also been argued that adolescents may perceive that they are being controlled and judged by using these passive assessment devices, and some authors have expressed concern about how sensors may negatively affect the doctor–patient relationship [20]. Furthermore, the sensors are designed to assess isolated brace compliance, but the assessment of other important psychosocial variables related to the brace (e.g., irrational beliefs, intense unpleasant emotions, or lack of social support) cannot be evaluated with the sensors.

During the last decades, the use of the Internet and mobile devices in our daily activities has increased dramatically, especially in adolescents [21,22]. The widespread use of these technologies has reached the healthcare context to improve both physical and psychological functioning [23,24]. In the specific context of AIS, web and mobile devices have been postulated as a useful alternative to sensors in the diagnosis and monitoring of AIS. For example, mobile applications have been used as screening tools for AIS to measure the Cobb’s degrees of curvature, even more accurately than traditional hand-held scoliometers [25]. Another alternative to sensors has been a web-based system for assessing brace compliance, which was well accepted by the participants [26]. Some limitations of the literature on alternatives to sensors for IS assessment should be considered. First, the daily measurement of how braces can affect physical activities and psychological wellbeing has not yet been included in these monitoring systems. Second, to date, patients or parents have been requested to log into a web system and upload the brace usage data at least once a day. This system has some limitations compared to apps, as the former requires Internet access and cannot use specific triggers such as a push system to start the survey.

It might be reasonable to think that the inclusion of an app-based monitoring system in clinical settings can help overcome the barriers of traditional, retrospective face-to-face assessments, and electronic devices (i.e., sensors and web pages). In the present study, we aim to test the feasibility and clinical utility of a new app-based management method. With this app, we intend to improve the monitoring of brace adherence, as well as the psychological and physical status of patients with AIS. Regarding feasibility, this study aims to explore the extent to which patients (adolescents with IS) and physicians accept the use of an app for daily monitoring. In relation to clinical utility, the objective of this work is to test whether the app detects adherence problems with the brace and related side effects and the clinicians respond quickly to them. We anticipate that the implementation of the app will be feasible for AIS monitoring (high acceptability and adherence to the app). In addition, we expect to find some indicators of the potential usefulness of the app (e.g., rapid detection of brace adherence problems and psychological and physical complications that allow a quick response by the medical team).

## 2. Materials and Methods

The current study uses a single group open trial design. In this feasibility study, we will monitor the use of brace and several outcomes related to brace use (e.g., psychological and physical status in relation to the brace) in a sample of adolescents with IS who are cared for in the Physical Medicine and Rehabilitation Service of a public tertiary hospital in Spain (Vall d’Hebron University Hospital). All adolescents who meet the inclusion criteria will be offered a mobile application for scoliosis monitoring. This app-based monitoring involves a daily evaluation for 90 days (3 months). Additionally, the assessment protocol includes two measurement points: before the brace use (baseline) and after three months of brace use (end of the study). These assessments (e.g., baseline and end of study) will be conducted using the Qualtrics online platform. The Ethics Committee of the Vall d’Hebron University Hospital approved the study and all its procedures. The Ethics Committee mentioned above aims to protect the safety of clinical trial participants and to ensure that there are no deviations from the expected plan. This study was previously registered in Clinicaltrials.gov (NCT04881591) on 10 May 2021. A standard Protocol Items Recommendations for Interventional Trial (SPIRIT) was followed to inform the protocol of the present study (Appendix A).

### 2.1. Participants

The participants included in this feasibility study will be 40 adolescents with IS. Sample size has been calculated according to previous recommendations suggesting a minimum of 30 participants in feasibility and pilot studies [27] and a minimum of 15 participants in usability studies with devices used in healthcare settings [28]. Sample size calculation includes a conservative correction of 20% for attrition.

Inclusion criteria:

- Age between 10 and 18 years when the brace is prescribed, Risser 0–2 and, if female, either before menarche or less than 1 year after menarche
- Primary curve angles 25°–40°.
- No prior brace treatment.
- The patient has a mobile phone with an Android or iOS operating system.
- The patient has the physical ability to use the mobile application.
- The patient does not present a serious psychological and/or cognitive problem or language alterations.
- Signed informed consent.
- Exclusion criteria:
- Not having a mobile phone or having a mobile phone with incompatible characteristics (i.e., unable to download the app).
- The patient has cognitive impairment or language problems to understand the use of the app and/or answer its questions.
- The patient has a serious mental health or substance abuse problem.

### 2.2. Recruitment and Procedures

Participants will be adolescents attending the Physical Medicine and Rehabilitation Service at Vall d’Hebron University Hospital who have been prescribed a brace for their IS. Participants who meet inclusion criteria (including their parents if participants are under 18 years of age) will receive the study information sheet (see Appendix B). If they give their voluntary consent to participate, they will be asked to sign the written informed consent (see Appendix B). Once the diagnosis is made by a physician from the Physical Medicine and Rehabilitation Service, patients with IS are prescribed a brace. Patients are asked to obtain the brace from an orthopedic center within the following week and are asked to test the brace for a few hours daily, mostly overnight, for a couple of weeks. This test period is used to verify if adjustments to the brace are required before a final version of the brace is provided and prescribed full time. An on-site consultation at the Physical Medicine and Rehabilitation Service is set to make these adjustments. This is the time when the app is also downloaded because it is at this stage that the final version of the brace is prescribed full time and on-site appointments become less frequent.

**Figure 1:**
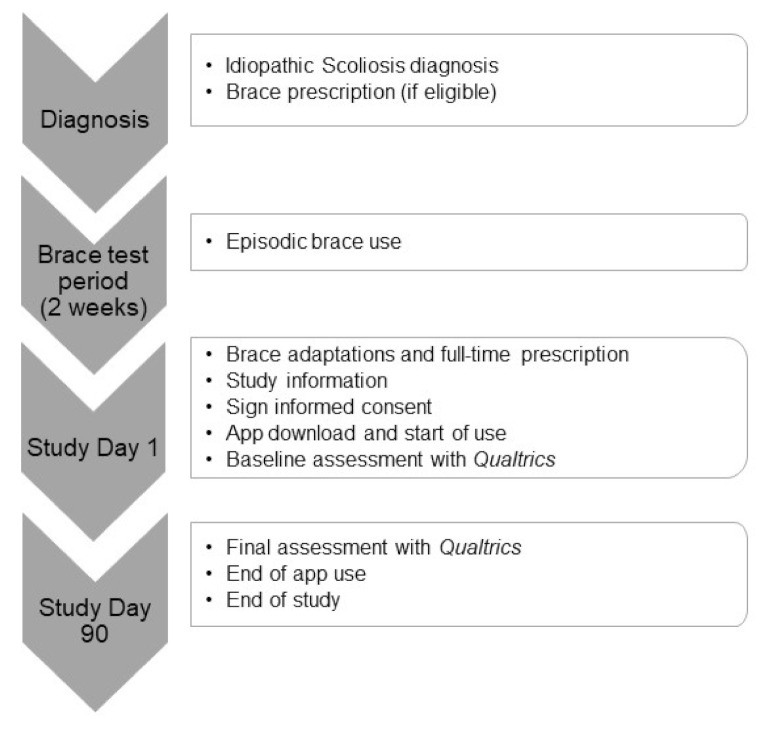
Study timeline.

This recruitment session is used to conduct the baseline assessment and download the app. A trainee hired for the present study will assign a unique anonymous alphanumeric code to patients (see Appendix C) that is linked to their identifying information (medical record number) in a separate document. This alphanumeric code is used to identify patients in both online and app assessments. The participants are asked to respond to a baseline assessment with Qualtrics. Their responses do not contain any identifying information. This evaluation includes sociodemographic data (i.e., age, sex, nationality), as well as scoliosis-related variables (i.e., stress, quality of life, perceived health, anxiety, and depressive symptoms). The trainee then helps the participants to download the app and explain to them how to use it daily for the next 90 days. After this period, a follow-up appointment is arranged to administer the end-of-study evaluation, again with Qualtrics. Figure 1 shows the study schedule.

### 2.3. Usual AIS Treatment + App-Based Ecological Momentary Assessment

In addition to the app, all adolescents receive the usual treatment for scoliosis that is provided in the Physical Medicine and Rehabilitation Service during the study period (90 days), that is, they are prescribed the daily use of a brace. In terms of monitoring, routine practice in the unit only includes a face-to-face assessment 3 months after the final version of the brace is prescribed, which means that problems with this final version of the brace are traditionally assessed retrospectively. Therefore, to minimize the problems associated with relying only on retrospective and episodic evaluations, the present study incorporates a daily app-based monitoring during 90 days since the start of brace treatment, which is a critical period in which the physical and psychological adaptation to full-time use of the brace might be more challenging.

The Pain Monitor app is a mobile application whose contents have been adapted to conduct ecological momentary assessments (EMA) in different health conditions. It has been previously validated in chronic pain conditions [29] and has received several awards since then (https://www.consalud.es/saludigital/145/premios-saludigital-2019-reconocen-mejores-iniciativas-tecnologia-sanitaria_60524_102.html, accessed on 11 July 2021). It is currently available for free in the Android (https://play.google.com/store/apps/details?id=monitorinvestigacion.code, accessed on 3 July 2021) and iPhone (https://apps.apple.com/es/app/monitor-de-dolor-multic%C3%A9ntrico/id1546241257, accessed on 3 July 2021) stores. The app evaluates important brace-related physical and psychological domains, namely side effects of the brace, pain intensity, mood, brace interference, brace adherence, avoidance behavior, discomfort, and social support. Following the procedure of a similar investigation [29], to create the EMA assessment protocol, a multidisciplinary team of psychologists and physicians revised the most frequent assessment tools in the AIS literature and selected the most representative constructs to create a representative, but short set of items (see a detailed description in Appendix D).

Participants respond daily to the questions in the app. They are prompted in the afternoon (at 7 pm) and have 2 h to respond (until 9 pm). If participants do not respond to the assessment by 8:30 p.m., a second reminder is sent. If participants access the application after 9 p.m., they are not allowed to complete the daily assessment, which is treated as missing data, and participants are instructed to access to the app the next day at the scheduled time. This is done to avoid backfilling and non-ecological assessment. EMA involves the assessment of the current or very recent status of patients [30]. If participants were allowed to respond to the daily assessments later than 9 p.m., this would compromise the ecological nature and comparability of the assessments. After 3 days of app non-compliance, the app sends a notification to the doctors, and they will call the patients to encourage compliance with the app.

Clinical alarms are generated in the app according to certain preset unwanted events (see Table 1). Every working day, the physicians will receive an anonymous report that will contain only the alphanumeric code described above, together with the description of the alarm. Next, the physicians access the document where the alphanumerical code is linked with the medical record number (Appendix C) to check the clinical history of the patients and decide the type of action necessary to solve the clinical alarm. Clinical alarms are not an emergency service and patients should use the services they usually use (i.e., emergency services, primary care, call to the Physical Medicine and Rehabilitation Service, etc.) in case they are faced with any symptom that worries them.

### 2.4. Physical Medicine and Rehabilitation Service Support According to Clinical Alarms

As reported in Table 1, each alarm generated by the app triggers a response from the medical team. This includes calling the patient (e.g., in the presence of intense and persistent pain) or sending the patient psychoeducational content by mail (see an example in Appendix E). This document has been created by a team of four psychologists trained in Cognitive Behavioral Therapy and Acceptance and Commitment Therapy for health problems and includes components of both types of interventions. Since the unit where the brace treatment is delivered does not have a psychologist, this content has been created to provide some psychological support when receiving alarms associated with psychological distress.

### 2.5. Outcome Measures

Primary outcome measures:

- The usability and acceptability of the app will be assessed by both patients (end users) and clinicians (relevant stakeholders). In the patients, this is evaluated both objectively and subjectively. To obtain an objective feasibility result, we calculate the adherence with the app by dividing the number of completed assessments by the number of planned evaluations and provide the response rate. To obtain a subjective measure of usability and acceptability, at the end of the study period (3 months after the first use of the app), we administer the System Usability Scale (SUS) [31] using an online survey tool that will be sent by mail (*Qualtrics*).
  In the clinicians, the acceptability of the new app-based monitoring method is evaluated using an assessment protocol developed for a similar earlier study [16]. As reported in Appendix D, this includes items that are consistent with the technology acceptance model [32], including perceived utility, acceptability, and intended use. This will be evaluated at the end of the study with the *Qualtrics* online survey tool anonymously.

Secondary outcomes measures:

Assessed by the Pain Monitorapplication (all once daily from 7 p.m. to 9 p.m.):

- Brace adherence: Ad hoc self-reported item: “When have you been able to wear the brace since you went to bed yesterday?” Response options cover all daily periods (morning only, afternoon only, only for sleeping, or any combination of these). To avoid bias due to socially desirable responses, honesty will be encouraged both during recruitment and in the informed consent.
- Treatment safety: An ad hoc question has been created including the most frequent side effects of brace use according to the literature [10,12] and the authors’ clinical expertise. These include: pain due to pressure, pain due to friction, excessive heat/sweating, movement difficulties, and being teased by peers or close ones.
- Clinical alarms: in addition to the assessment of brace adherence and side effects, several items were adapted from validated questionnaires (Appendix D) to assess pain intensity, unpleasant emotions, interference, avoidance, discomfort, and social support daily. Clinical alarms will be automatically generated and sent to the physicians by the app depending on the patients’ responses to these items (Table 1).

Assessed at baseline and at the end of the study with the *Qualtrics* online platform (see Appendix D for a detailed instrument description):

- Stress is measured with the Sobberheim Stress Questionnaire-Brace (BSSQ-Brace) [33,34].
- The Patients’ health-related quality of life is measured with the Italian Spine Youth Quality Of Life (ISYQOL) [35,36].
- Perceived health status is measured with the Scoliosis Research Society -22 (SRS-22) [37,38].
- Anxiety and depressive symptoms is measured with the Hospital Anxiety and Depression Scale (HADS) [39,40].

### 2.6. Ethics and Protection Data

The authors state that all the procedures included in this work comply with the ethical standard of the relevant national and institutional committees on human experimentation and with the Helsinki Declaration [41]. All the procedures described in this work were approved by the ethical committee of the Vall d’Hebron University Hospital. Modifications in the protocol are communicated to the ethical committee of the aforementioned hospital.

The responses provided by both assessment tools, namely Qualtrics and the app, are completely anonymous. Data collection and storage follow the Spanish law and data protection rules (“Ley Orgánica 3/2018, de 5 de diciembre, de Protección de Datos Personales y garantía de los derechos digitales”) [Spanish Data Protection Law], as well as Regulation (EU) 2016/679 of the European Parliament and of the Council of 27 April 2016 and Directive 95/46/EC (GDPR) on the protection of personal data and on the free movement of such data. Qualtrics^®^ is in accordance with the new General Data Protection Regulation (GDRP), is ISO 27,001 certified, and FedRAMP authorized. In addition, it allows correction, modification, and suppression of personal data in a permanent way (https://www.qualtrics.com/uk/platform/gdpr/ accessed on 11 May 2021).

If authorized by the patient in the informed consent form, the external researcher responsible for the study (Dr. Carlos Suso Ribera) has access to their personal information (i.e., phone number) to contact them and solve app-related technical issues (e.g., the app is not working or was deleted by mistake and needs to be reinstalled). These actions are recorded and reported as potential barriers for future implementation and acceptability of the new monitoring method. The study and its procedures are not associated with clinical complications or harmful effects. All participants may voluntarily suspend their participation in the study at any point. Their IS treatment and doctor–patient relationship will not be compromised by the discontinuation of the study.

## 3. Data Plan Analyses

Data are analyzed using intention-to-treat principles. Descriptive data (means, standard deviation, and frequencies) are reported for all study variables. These include usability and acceptability and response rates in the app (primary outcomes), together with brace adherence, the number of side effects, the number and type of clinical alarms generated by the app, stress, quality of life, perceived health status, and mood (secondary outcomes). Statistical differences between completers and non-completers are calculated. Completers are those who respond to at least 85% of the requested daily assessments, which is the average completion rate in EMA revealed in past meta-analytic research [42]. Adolescents who provide less than 85% responses to the app are considered non-completers.

Regarding feasibility, app adherence is calculated by dividing the number of responses registered in the app and the number of assessments programmed in the app (90 assessments, once daily during the whole study).

In relation to the clinical utility of the app, brace adherence is calculated by dividing the number of hours of use reported by the prescription they were given, as suggested in previous research [43]. Low brace adherence serves as a clinical alarm (Table 1). Changes in the remaining secondary measures (i.e., stress, perceived quality of life, perceived health status, and mood) from baseline to end of study are also explored, although it is expected to find minimal changes because these are not the main aim of the present study.

The results derived from the statistical analyses described above will be reported in the form of tables, graphs, and flowcharts. All the analyses are performed separately by the lead researcher and an independent researcher. An interim analysis is planned at the end of the study once 50% of the total sample has been evaluated. All coauthors and the Ethics Committee of the Vall d’Hebron University Hospital have access to these analyses and are allowed to participate in the decision to terminate the trial. The database is available under reasonable request for any researcher who requests it. Personal information from the participants is not included in this data set. The results from this feasibility study will be published anonymously in international journals and conferences.

## 4. Discussion

IS is a prevalent health condition that usually appears during adolescence due to the development of the spine that occurs at puberty [1,44]. IS can have a negative impact on psychological wellbeing, quality of life, disability, and physical health status, so early detection and treatment are essential [1,4,5,8]. The brace can be effective in the treatment of IS. However, its efficacy has also been questioned due to low adherence rates [9]. In an effort to improve brace compliance, studies have conducted retrospective assessments to investigate adherence and the factors that may negatively influence it [15]. Unfortunately, this methodology has a limited impact on the safety and effectiveness of treatment because assessment is conducted long after problems have arisen and is based on recall and memory, often leading to bias [17]. Additionally, face-to-face assessments require traveling (sometimes long distances), which increases costs and burden for patients (and their families). Electronic assessments can help overcome the limitations of retrospective face-to-face episodic assessments. Due to the widespread use of web and app-based devices in our daily activities, it seems feasible to integrate these devices into the daily monitoring of IS in adolescent populations. Some web and mobile applications have been used previously in detection of AIS and the assessment of adherence to the brace [25,26]. However, the assessment of the psychological and physical state of the patient, which could explain individual differences in adherence and provides important information for the safety and effectiveness of the treatment, has been never included in the daily monitoring of adolescents with IS.

The present protocol described a feasibility study whose aim is to test the usability, acceptability, and clinical utility of an app-based system for the monitoring of adolescents with IS who use a brace. Specifically, we hope to provide new insights into the feasibility of the app (i.e., app adherence and usability), as well as into its utility in detecting non-compliance with brace (treatment adherence), brace-related side effects like pain and interference in sleep (treatment safety), and psychological distress associated with wearing the brace (i.e., intense unpleasant emotions and poor social support). Regarding feasibility, we expect to find high compliance rates with the app-based monitoring system in the end users. Non-adherence to the app will generate clinical alarms, so the professional will be able to contact the participants to encourage them to interact with the app. For this reason, we expect to find low attrition rates during the study period (90 days). Additionally, in relation to the feasibility results, we anticipate that the participants will be very satisfied with the app in terms of acceptability and usability (i.e., “I needed to learn many things before I could start using the system”). Based on previous similar research [45,46], we also anticipate that the clinicians will be satisfied with the app in terms of perceived utility, low burden, and high intention to use. Regarding the clinical utility of the app, we also hypothesized that the app would allow early detection of low brace adherence, brace-related side effects, and psychological distress associated with brace use. Ultimately, we expect that the early detection of these symptoms with the app-based monitoring system will allow clinicians to quickly detect and solve unwanted events and thus provide safer and more effective treatment. To our knowledge, this is the first study to test the feasibility and clinical utility of telemonitoring adolescents with IS through a multidimensional app.

The present study has some limitations. Regarding the inclusion criteria, only adolescents with a smartphone will participate in the study. Therefore, a small proportion of patients may not be able to participate in the study. According to recent global statistics on smartphones, between 80% and 86% of adolescents between 12 and 17 years old own a smartphone [47,48]. Therefore, sample loss from not owning a smartphone is expected to be low. This information will be recorded and will be considered in the discussion on the feasibility of including this methodology for the monitoring of adolescents with IS. An additional shortcoming lies in the study’s ability to produce reliable findings regarding treatment effectiveness. Since this is a feasibility study and there will be no control group, it will be not possible to establish differences between groups and preliminary data on the clinical utility of the app should be interpreted with caution. However, note that the current study design is the preferred to provide information on the feasibility and potential clinical utility of implementing an app-based device for AIS monitoring. This type of study is important to justify whether a larger-scale randomized controlled trial, which is more expensive and time consuming, should be conducted [49]. Feasibility studies are crucial for novel interventions in general and for medical devices and health technology solutions in particular.

## 5. Conclusions

While we recognize the limitations mentioned above, if the app-based telemonitoring of adolescents with IS leads us to the hypothesized results in terms of feasibility and possible clinical utility, this study will have important clinical implications. Among the novelties of the present study is the implementation of an app-based EMA system for the multidimensional monitoring of adolescents with IS. An advantage of implementing this methodology includes the minimization of problems found in retrospective face-to-face episodic assessments (i.e., recall bias, poor monitoring that could compromise the safety of the treatment, and the burden on the physician associated with the time required for evaluations). Taking recall bias, ecological assessments are known to be more accurate than retrospective ones [17]. Therefore, the information obtained with these assessments is more reliable than that obtained from face-to-face appointments where the reliability of the data depends on the recall capacity of the patients [50]. Regarding treatment safety, the app will automatically detect the clinical alarms and send them to the physicians. Therefore, it will allow for timely detection of adverse brace-related symptoms and prompt management. Consequently, the app-based EMA will allow clinicians to provide more efficient and personalized treatments. Finally, and in relation to the burden of assessment, the app monitoring system will allow doctors to receive alarms passively, without the need to actively call patients at random moments during treatment and without patients having to travel to clinics. In fact, the entire monitoring process, as explained in this text, can be performed in an automated way with very little burden for the professionals.

In short, we hope that this new passive telemonitoring method will reduce the current burden on health care services, as doctors will contact patients only when necessary rather than routinely calling patients to assess their physical and psychological functioning. Last but not least, because alarms will occur on an individual level, treatments will become more personalized as specific responses and adaptations will be possible due to the variety of brace-related physical and psychological dimensions evaluated in the app.

## Figures and Tables

**Table 1:** Pre-specified clinical alarms detected by the mobile application.

Domain	Clinical Alarms	Recommendations ^1^
Side effects		
Pressure pain	3 consecutive days	Call the patient
Friction pain	3 consecutive days	Call the patient
Excessive heat/sweating	7 consecutive days	Call the patient
Movement difficulties	3 consecutive days	Call the patient
Teased by peers/relatives	4 consecutive days	Psychoeducation
Pain		
Pain intensity	≥3 during 3 consecutive days	Call the patient
Emotions		
Sadness	5 consecutive days	Psychoeducation
Anxiety	5 consecutive days	Psychoeducation
Anger	5 consecutive days	Psychoeducation
Shame	5 consecutive days	Psychoeducation
Overwhelm	5 consecutive days	Psychoeducation
Frustration	5 consecutive days	Psychoeducation
Interference		
Sleeping	3 consecutive days	Call the patient
Basic movements	3 consecutive days	Call the patient
Relationships (friends)	5 consecutive days	Psychoeducation
Relationships (relatives)	5 consecutive days	Psychoeducation
Leisure activities	5 consecutive days	Psychoeducation
Academic activities	5 consecutive days	Psychoeducation
Mood	5 consecutive days	Psychoeducation
Dressing	5 consecutive days	Psychoeducation
Self-image	5 consecutive days	Psychoeducation
Motivation for going out	3 consecutive days	Psychoeducation
Avoid talking about the brace	≥7 during 7 consecutive days	Psychoeducation
Avoid activities/being with others	≥5 during 5 consecutive days	Psychoeducation
Overall physical discomfort	≥5 during 5 consecutive days	Call the patient
Brace adherence		
Morning only (8–14 h)	7 consecutive days	Call the patient
Afternoon only (14–19 h)	7 consecutive days	Call the patient
Sleeping only (last night)	7 consecutive days	Call the patient
Morning and afternoon only	7 consecutive days	Call the patient
Sleeping and morning	15 consecutive days	Call the patient
Sleeping and afternoon	15 consecutive days	Call the patient
No use of the brace	3 consecutive days	Call the patient
Poor social support		
Friends	5 consecutive days	Psychoeducation
Family	5 consecutive days	Psychoeducation
Teachers	5 consecutive days	Psychoeducation
Other people	5 consecutive days	Psychoeducation

^1^ The physician may change the recommended action according to the patient’s condition.

## Data Availability

Data availability is not applicable to this article as no new data were created or analyzed in this study.

## Appendix A. SPIRIT 2013 Checklist: Recommended Items to Address in a Clinical Trial Protocol and Related Documents

**Section/Item**	**Item Nº**	**Description**	**Addressed on Page Number**
Administrative Information			
Title	1	Descriptive title identifying the study design, population, interventions, and, if applicable, trial acronym	1
Trial registration	2a	Trial identifier and registry name. If not yet registered, name of intended registry	4
	2b	All items from the World Health Organization Trial Registration Data Set	1
Protocol version	3	Date and version identifier	4
Funding	4	Sources and types of financial, material, and other support	11
Roles and responsibilities	5a	Names, affiliations, and roles of protocol contributors	1,11
	5b	Name and contact information for the trial sponsor	11
	5c	Role of study sponsor and funders, if any, in study design; collection, management, analysis, and interpretation of data; writing of the report; and the decision to submit the report for publication, including whether they will have ultimate authority over any of these activities	11
	5d	Composition, roles, and responsibilities of the coordinating center, steering committee, endpoint adjudication committee, data management team, and other individuals or groups overseeing the trial, if applicable	4–5
Introduction			
Background and rationale	6a	Description of research question and justification for undertaking the trial, including summary of relevant studies (published and unpublished) examining benefits and harms for each intervention	1–3
	6b	Explanation for choice of comparators	NA
Objectives	7	Specific objectives or hypotheses	2
Trial design	8	Description of trial design including type of trial (e.g., parallel group, crossover, factorial, single group), allocation ratio, and framework (e.g., superiority, equivalence, noninferiority, exploratory)	4
Methods: Participants, interventions, and outcomes			
Study setting	9	Description of study settings (e.g., community clinic, academic hospital) and list of countries where data will be collected. Reference to where list of study sites can be obtained	4–6
Eligibility criteria	10	Inclusion and exclusion criteria for participants. If applicable, eligibility criteria for study centers and individuals who will perform the interventions (e.g., surgeons, psychotherapists)	4
Interventions	11a	Interventions for each group with sufficient detail to allow replication, including how and when they will be administered	4–7
	11b	Criteria for discontinuing or modifying allocated interventions for a given trial participant (e.g., drug dose change in response to harms, participant request, or improving/worsening disease)	8–9
	11c	Strategies to improve adherence to intervention protocols, and any procedures for monitoring adherence (e.g., drug tablet return, laboratory tests)	5–6,9
	11d	Relevant concomitant care and interventions that are permitted or prohibited during the trial	5–7
Outcomes	12	Primary, secondary, and other outcomes, including the specific measurement variable (e.g., systolic blood pressure), analysis metric (e.g., change from baseline, final value, time to event), method of aggregation (e.g., median, proportion), and time point for each outcome. Explanation of the clinical relevance of chosen efficacy and harm outcomes is strongly recommended	7–8, Appendix D
Participant timeline	13	Time schedule of enrolment, interventions (including any run-ins and washouts), assessments, and visits for participants. A schematic diagram is highly recommended (see Figure 1)	5
Sample size	14	Estimated number of participants needed to achieve study objectives and how it was determined, including clinical and statistical assumptions supporting any sample size calculations	4
Recruitment	15	Strategies for achieving adequate participant enrolment to reach target sample size	4
Methods: Assignment of interventions (for controlled trials)			
Allocation:			
Sequence generation	16a	Method of generating the allocation sequence (e.g., computer-generated random numbers), and list of any factors for stratification. To reduce predictability of a random sequence, details of any planned restriction (e.g., blocking) should be provided in a separate document that is unavailable to those who enroll participants or assign interventions	NA
Allocation concealment mechanism	16b	Mechanism of implementing the allocation sequence (e.g., central telephone; sequentially numbered, opaque, sealed envelopes), describing any steps to conceal the sequence until interventions are assigned	NA
Implementation	16c	Who will generate the allocation sequence, who will enroll participants, and who will assign participants to interventions	4-5
Blinding (masking)	17a	Who will be blinded after assignment to interventions (e.g., trial participants, care providers, outcome assessors, data analysts), and how	NA
	17b	If blinded, circumstances under which unblinding is permissible, and procedure for revealing a participant’s allocated intervention during the trial	NA
Methods: Data collection, management, and analysis			
Data collection methods	18a	Plans for assessment and collection of outcome, baseline, and other trial data, including any related processes to promote data quality (e.g., duplicate measurements, training of assessors) and a description of study instruments (e.g., questionnaires, laboratory tests) along with their reliability and validity, if known. Reference to where data collection forms can be found, if not in the protocol	9
	18b	Plans to promote participant retention and complete follow-up, including list of any outcome data to be collected for participants who discontinue or deviate from intervention protocols	5-7
Data management	19	Plans for data entry, coding, security, and storage, including any related processes to promote data quality (e.g., double data entry; range checks for data values). Reference to where details of data management procedures can be found, if not in the protocol	8-9
Statistical methods	20a	Statistical methods for analyzing primary and secondary outcomes. Reference to where other details of the statistical analysis plan can be found, if not in the protocol	9
	20b	Methods for any additional analyses (e.g., subgroup and adjusted analyses)	NA
	20c	Definition of analysis population relating to protocol non-adherence (e.g., as randomized analysis), and any statistical methods to handle missing data (e.g., multiple imputation)	9
Methods: Monitoring			
Data monitoring	21a	Composition of data monitoring committee (DMC); summary of its role and reporting structure; statement of whether it is independent from the sponsor and competing interests; and reference to where further details about its charter can be found, if not in the protocol. Alternatively, an explanation of why a DMC is not needed	4
	21b	Description of any interim analyses and stopping guidelines, including who will have access to these interim results and make the final decision to terminate the trial	9
Harms	22	Plans for collecting, assessing, reporting, and managing solicited and spontaneously reported adverse events and other unintended effects of trial interventions or trial conduct	6-9
Auditing	23	Frequency and procedures for auditing trial conduct, if any, and whether the process will be independent from investigators and the sponsor	NA
Ethics and dissemination			
Research ethics approval	24	Plans for seeking research ethics committee/institutional review board (REC/IRB) approval	4, 11
Protocol amendments	25	Plans for communicating important protocol modifications (e.g., changes to eligibility criteria, outcomes, analyses) to relevant parties (e.g., investigators, REC/IRBs, trial participants, trial registries, journals, regulators)	8
Consent or assent	26a	Who will obtain informed consent or assent from potential trial participants or authorized surrogates, and how (see Item 32)	4-5
	26b	Additional consent provisions for collection and use of participant data and biological specimens in ancillary studies, if applicable	NA
Confidentiality	27	How personal information about potential and enrolled participants will be collected, shared, and maintained in order to protect confidentiality before, during, and after the trial	8-9
Declaration of interests	28	Financial and other competing interests for principal investigators for the overall trial and each study site	11
Access to data	29	Statement of who will have access to the final trial dataset, and disclosure of contractual agreements that limit such access for investigators	9
Ancillary and post-trial care	30	Provisions, if any, for ancillary and post-trial care, and for compensation to those who suffer harm from trial participation	8-9
Dissemination policy	31a	Plans for investigators and sponsor to communicate trial results to participants, healthcare professionals, the public, and other relevant groups (e.g., via publication, reporting in results databases, or other data sharing arrangements), including any publication restrictions	9
	31b	Authorship eligibility guidelines and any intended use of professional writers	NA
	31c	Plans, if any, for granting public access to the full protocol, participant-level dataset, and statistical code	9
Appendices			
Informed consent materials	32	Model consent form and other related documentation given to participants and authorized surrogates	Appendix B
Biological specimens	33	Plans for collection, laboratory evaluation, and storage of biological specimens for genetic or molecular analysis in the current trial and for future use in ancillary studies, if applicable	NA

## Appendix B. Informed Consent

Project title: Improving the quality and safety of adolescent idiopathic scoliosis treatment

Principal investigators: Judith Sanchez Raya

Service: Physical Medicine and Rehabilitation Service at Vall d’Hebron University Hospital.

Promoters: Universitat Jaume I and Vall d’Hebron University Hospital

Aims: the main objective of this study to monitor adolescents with idiopathic scoliosis (IS) under brace treatment. Specifically, we will use the free Pain Monitor app to detect the problems you may experience with brace treatment as early as possible.

With this document we want you to have the correct and sufficient information to decide whether you want to participate in this study. Read this informed consent carefully. We will answer any question you may have.

Study procedures: In this study we will propose all adolescent patients with IS attending the Physical Medicine and Rehabilitation Service of the Vall d’Hebron University Hospital to use the mobile application. The Physical Medicine and Rehabilitation Service’s medical team will offer you to participate during your medical appointment. If you accept, we will ask you and your parent/legal tutor to sign an informed consent form. Before downloading the app, we will ask you to answer a few questions about your health on the *Qualtrics* online platform. We will require your email address to send you the survey link. In *Qualtrics,* you will be asked to use the same random code that we will give you for the app. Thus, your answers will always be anonymous. We will then help you download the app into your mobile phone and show you how to use it. Due to the characteristics of the study, only patients with their own mobile phone will be able to participate. Three months later, we will ask you to answer the same questions you answered in the first survey on the *Qualtrics* platform. We will send you the assessment link again. This will be the end of the study, but the medical team will continue to monitor you.

It is possible that your answers in the app will generate an alarm that will be sent to the medical team during the next working day. In that case, if the medical team thinks this is necessary, they may contact you. However, as alarms do not arrive instantly and will not always be answered, if you are worried about something related to your treatment, do not wait for them to contact you. Talk to your parents or legal tutors and do what you would normally do, such as going to the emergency service, visiting your primary care doctor or calling the Physical Medicine and Rehabilitation Service.

Protection data: We will store all your answers securely both in the app and in *Qualtrics*. This will be done by the research team. They will follow the current security laws to protect your data (Ley Orgánica 3/2018, de 5 de diciembre, de protección de datos personales y garantía de los derechos digitales [Spanish law]). Your answers will not be linked to any of your personal information (name, phone number, or address).

Only the medical team participating in this study will be able to identify you with your answers using a document that the principal investigator (Dra. Judith Sanchez Raya) will keep securely. Of all the staff outside the Physical Medicine and Rehabilitation Service, only the study promoter (Dr. Carlos Suso Ribera), who is in charge of the study, will be able to know your telephone number if you give him your consent on the informed consent form. This is important so that he can call you if he detects technical problems with the app (in no other case). It is possible that the medical team may also call you during the study (for example, if they receive an alarm from the app).

This study will follow the strictest rules (Organic Law 3/2018 of 5 December and regulation 2016/679 of the European Parliament and of the Council) to ensure that we treat your data in the most appropriate way and following the regulation. We will not ask you for more personal data than necessary. Under no circumstances your personal details will be given to anyone outside the study, except in the case of a medical emergency that justifies it or if we are legally bound to do so.

Please note that the data from the app will not be included in your clinical history, although the data from the *Qualtrics* survey could be included. In any case, if you have any questions about your answers in the app or in *Qualtrics* (for example, if you want to see them, have them or change them), please contact the study promoter, (Dr. Carlos Suso Ribera), or the Data Protection Delegate at the Universitat Jaume I (Dra. Diana Castilla, dolorcronico@uji.es).

The Legal Unit of the Vall d’Hebron University Hospital will resolve your doubts, complaints, clarifications and suggestions by e-mail: (lopd@vhir.org), or by post: (Paseo Vall d’Hebrón 119–129, Edificio Mediterránea 2^a^ Planta, 08035 Barcelona). You should be aware that data cannot be deleted even if you stop participating in the study in order to guarantee the validity of the research. You can also contact the Data Protection Agency if you have any further questions.

You have the right to be informed of any important information about your health that is detected in the study. You have the right to choose whether or not you want this information.

Voluntary participation and right to withdraw the informed consent: You should be aware that your participation in this study is voluntary. You may decide not to participate or to change your decision and withdraw your consent at any time. This will not negatively affect the relationship with your doctor or your treatment.

Informed Consent: Project title: Improving the quality and safety of adolescent idiopathic scoliosis treatment

I______________________________________________________________.

I have read the informed consent given to me.

I have been able to ask questions about the study.

I have spoken to: Dra. Judith Sánchez Raya.

I understand that my participation is voluntary and that my data cannot be associated with an identified or identifiable person because the information that identifies me in the app and *Qualtrics* has been replaced by a code.

I understand that my answers to the app are not stored in any official medical record and my answers in *Qualtrics* may be included at the discretion of the medical team. Therefore, if you have any questions about your answers, please contact the study promoter (Dr. Carlos Suso Ribera; susor@uji.es).

I understand that I can withdraw from the study:

Whenever I want

Without having to provide detailed explanations

Without this negatively influencing my medical care

I voluntarily agree to participate in the study.

I agree that the physicians responsible for this study may contact me in the future if it is deemed appropriate to add new data to the data collected:

Yes

No

I agree that the promoter of the study and professor at the Universitat Jaume I, may contact me by phone if an app malfunctioning problem is detected:

Yes

No

Date and signature of the participant/relative/legal tutor	Date and signature of the researcher

Informed Consent Withdraw

I______________________________________________________________or familiar/relative/legal tutor (if applicable) of the patient ___________________________ (name and surname) revoke the above signed informed consent to participate in the study.

This revocation of the informed consent means that from the date on which this consent is signed, no further medical data may be collected without prejudice to the conservation of the data resulting from the research previously carried out.

Date and signature of the participant/relative/legal tutor

## Appendix C. Alphanumeric Code Linked with Identifying Information

**Number of Participant**	**Alphanumeric Code**	**Medical Registry Number**
1	1ESCVH0001	9999999
2	1ESCVH0002	9999998
3	1ESCVH0003	9999997
4	1ESCVH0004	9999996
5	1ESCVH0005	9999995

## Appendix D. Assessment Protocol

1. **Items in the *Qualtrics* Online Platform**

1.1 Items assessed once, in the baseline assessment

Please select your age (in years)

10 (1)… 18(9)

Please indicate your sex:

Male

Female

Intersexual

Please select the country in which you born

Spain (163)… Zimbabwe (1357)

1.2 Questionnaires administered twice at baseline (before the brace and app use) and at the end of the study (after 3 months of brace and app use):

- Sobberheim Stress Questionnaire (BSSQ brace) [33,34]. It contains eight items assessing psychological stress produced by brace in adolescents with IS (i.e., *I find it hard to show my back in public*”). Response use a 4-points Likert scale (0 = “most stress” to 3 = “least stress”). Total scores range from 0 to 24. Higher total scores indicate less stress. Cut-offs proposed in the original versions are: 0–8 (severe stress), 9–16 (moderate stress), and 17–24 (mild stress) [34].
- Italian Spine Youth Quality Of Life (ISYQOL) [35,36]. The scale is composed of 20 items. Some of them assess quality of life with respect to back problems and can be administered to all patients with spinal deformities (spine health domain = 13 items; “*Despite your back problem, do you life a happy life?*”). The remaining items are specifically designed for patients wearing a brace (brace domain = 7 items; “*It is uncomfortable to wear your brace?*”). Responses use a 3-point Likert scale (0 = “never”; 1 = “sometimes”; 2 = “often”). Items 5, 6, 10, and 13 are reverse coded. Total scores are converted into percentages [35]. Final scores range from 0% to 100%, where higher percentages indicate greater quality of life.
- Scoliosis Research Society-22 (SRS-22) [37,38]. This questionnaire is composed of 22 items that evaluate 5 scoliosis domains, namely function/activity (5 items; “*What is your current level of activity?*”), pain (5 items; “*Which one of the following best describes the amount of pain you have experienced over the last month*”), self-image/appearance (5 items; “*How do you look in clothes?*”), mental health (5 items; “*Have you felt calm and peaceful during the last 6 months?*”), and satisfaction with the treatment (2 items; “*which one of the following best describes your pain medication use for back pain?*”). Response scales range from 1 = “Severe/All of the time/Very unhappy/Bedridden/Very bad/Very often/Very poor/None of the time/Severely/Very unsatisfied/Definitely no” to 5 = “None/None of the time/Very happy/Full activities without restriction/Very good/Never/Very good/All the time/Very satisfied/Definitely yes”. Higher scores indicate better perceived health status.
- Hospital Anxiety and Depression Scale (HADS) [39,40]. The HADS consists of 14 items that assess anxiety (seven items; “*I feel tense or wound up*”) and depression (seven items; “*I still enjoy the things I used to enjoy*”). Each item is rated according to a 4-point Likert scale ranging from 0 = “as much as I always do” to 3 = “not at all”. Total scores range from 0 to 21 and higher scores represent higher anxiety and depressive symptoms. Cut-offs are stablished as follows: 0–7 (non-cases), 8–10 (doubtful case), and 11–21 (definite case) [39].
- System Usability Scale (administered only at the end of the study). The SUS evaluates whether a system is considered to be simple to use and useful (e.g., “*I needed to learn many things before I could start using the system”*). It is responded to according to a 5-point Likert scale (1 = “completely disagree” to 5 = “completely agree”). The score of half of the items has to be reverted so that higher scores indicate higher usability and acceptability. Then total score is multiplied by 2.5 so that final scores range from 0–100.

1.3 Items used to evaluate app acceptability in the clinicians (all item responses have a 0–4 ranging from “Completely disagree” to “Completely agree”)

To what extent the app was useful for IS management?

To what extent the app increases IS treatment safety?

To what extent the app increases IS treatment effectiveness?

To what extent the app gives me comfort when managing IS?

To what extent the app is useful for me as a professional?

To what extent the app can be useful for patients with IS?

To what extent the app with alarms is something I want to use in the future?

To what extent the app without alarms is something I want to use in the future?

To what extent the app alarms impacted daily job burden?

To what extent the app has an impact on burden (help patient downloading the app)?

2. **Items Assessed with the Pain Monitor Mobile Application**

A number of items were adapted from or developed considering well-established questionnaires (e.g., BSSQ, ISYQOL, SRS-22, HADS) in order to assess relevant brace-related domains namely side effects of the brace, avoidance behavior, discomfort, and social support.

Items assessed daily at evening

Please indicate if any of these possible consequences of the brace have been difficult for you to bear TODAY. You may select more than one option: (*Inspired by the SRS-22 and the ISYQOL*)

Pressure pain

Friction pain

Excessive heat/sweating

Movements difficulties

Teasing by peers or relative ones

None of the above have difficult for me today

Please indicate the intensity of your pain TODAY. Indicate the average for the whole day between 0 and 10. (*Inspired by the SRS-22*)

0 No pain ---------10 Extreme pain

Please indicate if you feel some of these intense emotions TODAY due to bracing. You may select more than one option: (*Inspired by the SRS-22 and the HADS*)

Sadness

Anxiety

Anger

Shame

Overwhelm

Frustration

I did not feel any of these emotions intensely because of the brace

Please indicate if brace have interfered you in some of these aspects TODAY. You may select more than one option: (*Inspired by the SRS-22, the ISYQOL, and the BSSQ*)

Sleeping (last night)

Basic movements

Friends’ relationships

Family’ relationships

Leisure activities

Academic activities

Mood

Dressing

Self-image

Motivation for going out (classroom or leaving home)

None of the above have disturbed me today

When have you been able to wear the brace sin you went to bed yesterday? [Item created to assess de degree of nonadherence (mildly vs. severe nonadherent), as recommended by [51]

Only at morning (8–14 h approx)

Only at afternoon (14–19 h approx)

Only for sleeping (last night)

Today at morning and afternoon, but no for sleeping (last night)

Sleeping (last night) and this morning

Sleeping (last night) and this afternoon

Sleeping (last night) and all day

I was not able to wear the brace today

Please indicate how much have you avoided talking about your brace today. Rate from 0 to 10. (*Inspired by the BSSQ*)

0 I have not avoided it at all ------- 10 I have avoided it a lot

Please indicate how much have you avoided doing things you like or being with others to hide your brace today. Rate from 0 to 10 (*Inspired by the BSSQ*)

0 I have not avoided it at all ------- 10 I have avoided it a lot

Indicate from 0 to 10 the degree of discomfort with which you have worn the brace TODAY. (*Inspired by the ISYQOL*)

0 No discomfort ------- 10 Maximum discomfort

Select if you have lacked the support of any of these people regarding your brace TODAY. You may select more than one option: [*Inspired by the ISYQOL and the Brace Beliefs Questionnaire* [52]]

Friends

Family

Teachers

Other people

I felt supported today

## Appendix E. Response to Clinical Alarm

### Coping Recommendations When Wearing a Brace

Starting to wear a brace can be difficult. It may lead to questions by others (What is it that you are wearing?) or even rejection (This that you are wearing is very ugly). Of course, everyone would prefer not to wear a brace (similar to what happens with orthodontics). However, if we give a great deal of importance to the criticism of others or to being judged, the risk is that we will stop doing things that we care about because of this concern and fear that we will be negatively evaluated by others.

Remember that if you stop doing important things so that other people do not notice your brace, this will add to your psychological distress. In addition to the suffering associated with wearing a brace, a second suffering will then be added, that is, the suffering associated with stopping to participating and enjoying the things and the people you love.

If you have been quitting doing important things for you so that other people do not notice your brace or if you think that wearing a corset is affecting you a lot, we recommend:

- Remember that the use of a brace is temporary.
- Be compassionate with yourself. Suffering is normal in this situation. Talk about it with the people you trust.
- Reward yourself. Do not isolate yourself from certain people or activities, as this will make things even harder for you. Reconnect with what you like by accepting the brace as a difficulty that you can live with. Try experiencing pleasant emotions with the activities you like.
- Do not avoid interacting with people or doing activities due to brace or the fear will become bigger and bigger. You can change and go back to enjoying the things you like (of course, wearing the corset makes it less pleasant, but still worth it).
- Some discomfort associated with wearing the brace is worth what you earn by participating in the things you like.
- Do not stop doing things that you like because they can judge you. Connect with what you like about your activities, with the joy. Accept that, although the brace makes things more difficult, the activities you like continue to be pleasant.
- Go ahead and participate in activities that you like, activities that can generate well-being even if your head tells you that they will judge you or that you will have a hard time. In the end, you may experience an uncomfortable moment (someone who asks about the brace or judges you), but it will only be episodic. Going through that short difficult moment will allow you to enjoy the rest of the activity.
- It is possible that, when you plan to do activities, your head shares difficult thoughts with you. These may include things like “they will see my corset”, “they will think I am different”, “they will judge me…”. In those moments, it can be useful to remember the reasons why you liked this activity before wearing the brace and remember the good times you have spent doing it.
- Allow yourself not to talk about it when you do not feel like it. Others have the right to ask, just as you have the right to not answering.
- Wearing a brace is not pleasant, but it is something that will help your health. It is normal that you do not like it and that you prefer not to wear it. However, accepting that now is the time to make this effort for your future well-being and understanding (and welcoming) that this entails difficulties and discomfort will allow you to better cope with it.
- Allows the corset to be there. Even allow it to be noticed externally. Instead of fighting against it so that it is not noticed, allowing it to be there and telling yourself that “nothing devastating happens if it is noticed” will help you to cope better. Of course, it would be more comfortable not to wear a brace, but you can wear it and at the same time do the things you like if you give up the fight against it.
- Enjoy who you are now. Do not wait to be that almost unreachable hypothetical perfect person to get involved in your life and do the things that give you a sense of fulfillment.
- Think that, even when somebody criticizes or judges you, that is temporary. You do not have to be liked by everyone at all times and you can do things that you like even when someone judges you. That judgment is a discomfort that you can carry along with you in the process of living a meaningful life that is worth living.
- Remember that the important thing is not to do things perfectly or without criticism. The important thing is to do things with interest and involvement, understanding and welcoming that things will not always be easy and that you will not always do things perfectly.
- Think about what YOU want to get out of life, not just what OTHER PEOPLE expect.
- Talk to yourself in a compassionate and kind manner. All of us go through bad times and make mistakes. What is important is to realize this and to be willing to make changes.
- Take care of yourself and give yourself messages of appreciation when you make efforts.
